# Left Frontal White Matter Links to Rhythm Processing Relevant to Speech Production in Apraxia of Speech

**DOI:** 10.1162/nol_a_00075

**Published:** 2022-09-22

**Authors:** Rose Bruffaerts, Jolien Schaeverbeke, Ahmed Radwan, Manon Grube, Silvy Gabel, An-Sofie De Weer, Eva Dries, Karen Van Bouwel, Timothy D. Griffiths, Stefan Sunaert, Rik Vandenberghe

**Affiliations:** Laboratory for Cognitive Neurology, Department of Neurosciences & Leuven Brain Institute, Katholieke Universiteit Leuven, Leuven, Belgium; Neurology Department, University Hospitals Leuven, Leuven, Belgium; Computational Neurology, Experimental Neurobiology Unit (ENU), Department of Biomedical Sciences, University of Antwerp, Antwerp, Belgium; Biomedical Research Institute, Hasselt University, Diepenbeek, Belgium; Translational MRI, Department of Imaging and Pathology & Leuven Brain Institute, Katholieke Universiteit Leuven, Leuven, Belgium; Biosciences Institute, Medical School, Newcastle University, Newcastle-upon-Tyne, UK; BIFOLD, Technische Universität Berlin, Germany; Department of Psychology, Ashoka University, India; Radiology Department, University Hospitals Leuven, Leuven, Belgium

**Keywords:** speech production, rhythm, apraxia of speech, structural MRI, psychoacoustics

## Abstract

Recent mechanistic models argue for a key role of rhythm processing in both speech production and speech perception. Patients with the non-fluent variant (NFV) of primary progressive aphasia (PPA) with apraxia of speech (AOS) represent a specific study population in which this link can be examined. Previously, we observed impaired rhythm processing in NFV with AOS. We hypothesized that a shared neurocomputational mechanism structures auditory input (sound and speech) and output (speech production) in time, a “temporal scaffolding” mechanism. Since considerable white matter damage is observed in NFV, we test here whether white matter changes are related to impaired rhythm processing. Forty-seven participants performed a psychoacoustic test battery: 12 patients with NFV and AOS, 11 patients with the semantic variant of PPA, and 24 cognitively intact age- and education-matched controls. Deformation-based morphometry was used to test whether white matter volume correlated to rhythmic abilities. In 34 participants, we also obtained tract-based metrics of the left Aslant tract, which is typically damaged in patients with NFV. Nine out of 12 patients with NFV displayed impaired rhythmic processing. Left frontal white matter atrophy adjacent to the supplementary motor area (SMA) correlated with poorer rhythmic abilities. The structural integrity of the left Aslant tract also correlated with rhythmic abilities. A colocalized and perhaps shared white matter substrate adjacent to the SMA is associated with impaired rhythmic processing and motor speech impairment. Our results support the existence of a temporal scaffolding mechanism structuring perceptual input and speech output.

## INTRODUCTION

Recent neurophysiological evidence proposes a central role for rhythm processing in speech production and speech perception (Poeppel & Assaneo, 2020). Here, we examine this link in individuals with the non-fluent variant (NFV) of primary progressive aphasia (PPA) with apraxia of speech (AOS). The rhythmicity of speech originates at the suprasegmental level as a result of the metrical frame of words containing multiple segments or syllables (Aichert et al., 2016). Speech rhythm also enhances perception: Speech perception is optimal between 2 and 8 Hz (Ghitza & Greenberg, 2009), and the auditory cortex is tuned to these frequencies (Boemio et al., 2005). The relationship between the neural processes supporting speech rhythm and speech perception is under debate. Grube et al. (2012) have proposed a “temporal scaffolding mechanism,” a common neurocomputational mechanism which structures both perceptual input and speech production, in time. Specifically, they observed that abilities to detect timing differences in meaningless auditory stimuli correlated with phonological abilities (reading, repetition). This correlation between rhythm and phonological processing may reflect their relevance in parsing the speech stream and extracting suprasegmental attributes. Patients with NFV and AOS represent a unique opportunity to test the temporal scaffolding hypothesis because impaired speech production co-occurs with a perceptual deficit in this population (Goll et al., 2010; Grube et al., 2016). Clinically, NFV patients present with effortful, non-fluent speech with simplified structures of their utterances (Grossman, 2018). A substantial number of NFV cases also demonstrate AOS (Duffy et al., 2014), a motor speech disorder that leads to phonetic errors, trial-and-error articulation (Ogar et al., 2007), slow speech rates, syllable segmentation, and lengthened intersegment durations (Josephs et al., 2012). Vowel lengthening is often observed in AOS (Ballard et al., 2014; Josephs et al., 2012). Typical AOS-related changes in speech timing consist of relative lengthening of the first unstressed vowel compared to the second stressed vowel (Duffy et al., 2017), impacting the suprasegmental level of speech while often still respecting the representation of stress. Etiologically, AOS is caused by impaired motor planning of movements for speech (Duffy et al., 2014; Grossman, 2018; Ogar et al., 2007). Another motor speech impairment, dysarthria, is also observed in NFV with AOS in an estimated 20–50% of patients (Duffy et al., 2014). The dysarthria co-occurring with AOS is mainly of the spastic or hypokinetic type (Duffy et al., 2014). It is clear that the co-occurrence of these two motor speech disorders (AOS and dysarthria) complicates the assessment of the phenotype, as dysarthria in itself may impact neurolinguistic test scores and, especially in the case of ataxic dysarthria, also the speech rhythm.

Co-occurring auditory perceptual abnormalities have been documented before in NFV with AOS. Compared to controls and patients with the semantic variant (SV) of PPA, patients with NFV were less able to discriminate between sounds with different spectral shapes (Goll et al., 2010). Compared to controls and SV, NFV were also impaired at detecting changes in a rhythmic pattern, which relies on the capacity to extract suprasegmental timing (Grube et al., 2016).

In a PPA cohort including 12 patients with NFV and AOS (Table 1) and 11 patients with SV (Table 2), we here study the neuroanatomical correlate of impaired auditory rhythmic processing in a search for supporting evidence for a temporal scaffolding mechanism. Grey matter atrophy is consistently found in NFV in the left opercular part (BA44) of the inferior frontal gyrus (IFG), insula, premotor and the supplementary motor areas (SMA) (Gorno-Tempini et al., 2011; Rogalski et al., 2011). The degree of atrophy in these regions correlates with markers of speech timing in patients with AOS (Ballard et al., 2014). SMA has been identified as a gray matter correlate of auditory rhythmic processing in healthy volunteers (Grahn & Brett, 2007; Grahn & Schuit, 2012) and NFV (Hardy, Agustus, Marshall, Clark, Russell, Bond, et al., 2017; Hardy, Agustus, Marshall, Clark, Russell, Brotherhood, et al., 2017). The correlational neuroimaging findings in NFV align well with recent neurophysiological evidence. For instance, oscillatory activity in IFG and motor regions has been linked to speech perception (Assaneo & Poeppel, 2018; Keitel et al., 2018; Magrassi et al., 2015). Left frontal white matter changes also contribute to impaired speech production in NFV (Canu et al., 2019; Galantucci et al., 2011; Mandelli et al., 2014), which motivated our decision to investigate whether white matter changes correlate with rhythmic abilities in NFV. We used two independent modalities: tensor-based deformations of the brain (deformation-based morphometry, DBM) and diffusion tensor imaging (DTI) combined with constrained spherical deconvolution-based tractography (CSD), which has been shown to improve tractography results even in low angular resolution data (Calamuneri et al., 2018). We opted for DBM rather than voxel-based morphometry (VBM) because automated segmentation in regions of abnormal grey and white matter might be unreliable, and because DBM allows visualization of changes in subcortical structures containing grey and white matter (Cardenas et al., 2007). DBM is also easier to interpret than VBM since it reflects atrophy without inference from other pathological white matter changes. Furthermore, we complement DBM with DTI. DTI is sensitive to white matter damage even at the individual level (Sajjadi et al., 2013), which is beneficial given the relative rarity of NFV. Damage of the left Aslant tract, which connects BA44 to medial frontal areas including the SMA (Catani et al., 2013), is considered specific for the NFV phenotype (Canu et al., 2019; Mandelli et al., 2014). We thus focused our DTI analysis on the left frontal Aslant tract. If a temporal scaffolding mechanism exists in the brain, i.e., a common neural substrate that structures auditory input and output in time, we would predict that there is an overlap in the atrophy patterns associated with impaired rhythmic processing and those previously linked to AOS.

**Table 1.** Characteristics of NFV patients.

**Case**	**6**	**13**	**15**	20*	21*	22*	23*	**25**	31*	**36**	**37**	**38**	**Cut-off**
Age	52	79	71	78	72	63	62	57	69	58	80	65	/
Sex	F	F	M	M	F	F	F	F	M	F	F	M	/
Education	17	8	15	17	12	15	12	10	18	12	10	16	/
CPM (/36)	31	**24**	**24**	30	**12**	32	31	/	29	**21**	**4**	35	<28
Dis. Dur.	2	5	1.5	2.5	2.5	5	2.5	3.5	1.5	4	4	3	/
BNT (/60)	58	**48**	55	**48**	**30**	**41**	**46**	**7**	**29**	52	**27**	57	<50
AAT rep 1 (/30)	28	29	30	29	**26**	30	28	**27**	**20**	28	**14**	30	<28
AAT rep 2 (/30)	**26**	**24**	**21**	30	**28**	30	30	29	30	**28**	**25**	30	<29
AAT rep 3 (/30)	**28**	**22**	29	**28**	**24**	30	30	**28**	29	**28**	**27**	29	<29
AAT rep 4 (/30)	**26**	**23**	30	29	**24**	30	29	**15**	29	28	**14**	30	<28
AAT rep 5 (/30)	**26**	**27**	30	30	**15**	30	28	**17**	28	**23**	**10**	30	<28
DS	6	**3**	4	6	**2**	4	5	**3**	7	5	**3**	4	<4
DIAS cons (/15)	/	/	/	**13**	/	15	**10**	14	**13**	**3**	**0**	15	<14
DIAS vow (/15)	/	/	/	15	/	15	**14**	**14**	**14**	15	**6**	15	<15
DIAS dia	/	/	/	103	/	77	**50**	**24**	115	**48**	**6**	**47**	<56
WEZT (/40)	/	/	/	**36**	/	**35**	**36**	**29**	**31**	**20**	**11**	38	<38
Dysarthria	−	−	**S**	−	−	−	**S**	−	**H**	−	**SH**	**H**	/
Extrapyr	−	−	**+**	−	−	**+**	**+**	**+**	**+**	−	−	−	/
CIT Spect	/	/	/	/	/	/	/	+	/	/	/	/	/
FDG-PET	SMA	L IFG	L Frontal	L IFG	Nl	/	Nl	/	Nl	L SMA	L Frontal	L SMA	/
Tau CSF	/	312	/	195	**424**	183	/	247	/	298	273	**530**	>367
Aβ42 CSF	/	1028	/	816	1060	865	/	1057	/	1320	1264	1139	<500
Neuropath	/	CBD	CBD	/	/	/	/	/	/	/	/	/	/

*Note*. Norms were calculated as 2 standard deviations below the mean in the age- and education-matched group of healthy controls (*n* = 29). CPM: Raven’s progressive matrices. Dis. Dur.: disease duration (years). BNT: Boston Naming test. AAT: Akense Afasie test repetition scores parts 1 to 5. DS: digit span forward. DIAS cons: Diagnostic Instrument for AOS (DIAS) repetition of consonants. DIAS vow: DIAS repetition of vowels. DIAS dia: DIAS diadochokinesis score. WEZT: grammaticality score on Verbs and Sentences Test. Dysarthria: if present, the type of dysarthria is indicated (S: spastic, H: hypokinetic, SH: mixed spastic-hypokinetic). Extrapyr: extrapyramidal signs upon examination. CIT Spect: [123I]ß-CIT (2ß-carbomethoxy-3ß-(4-iodophenyl)tropane) SPECT to evaluate the presynaptic dopaminergic system. FDG-PET: [18F]fluorodeoxyglucose PET, hypometabolism was observed in the SMA, IFG, or whole frontal lobe, either bilateral or on the left side (L). Cerebrospinal fluid (CSF) analysis of Aβ1-42 and total tau using ELISA. Neuropath: anatomopathological findings. *Also meets criteria for the mixed variant of PPA. NFV: non-fluent variant of primary progressive aphasia. Values outside the normal range are in bold.

**Table 2.** Characteristics of SV patients.

**Case**	**1**	**5**	**7**	**12**	**14**	**17**	**19**	**24**	**27**	**29**	**32**	**Cut-off**
Age	76	70	61	64	48	58	69	73	63	55	70	/
Sex	M	F	M	M	F	F	F	F	F	M	F	/
Education	14	12	17	8	12	10	7	14	13	14	17	/
CPM (/36)	29	**24**	36	29	35	36	34	28	34	34	35	<28
Dis. Dur.	6	1.5	3	3	6	2	5	1.5	1	0.5	9	/
BNT (/60)	**34**	**22**	**7**	**20**	**12**	**35**	**16**	**11**	**17**	**33**	**4**	<50
AAT rep 1 (/30)	30	30	29	28	30	30	30	30	30	30	30	<28
AAT rep 2 (/30)	**27**	29	29	**27**	30	30	**28**	29	**27**	30	30	<29
AAT rep 3 (/30)	30	30	30	30	30	30	29	30	**28**	30	30	<29
AAT rep 4 (/30)	30	30	28	29	30	28	30	30	28	30	30	<28
AAT rep 5 (/30)	30	28	**25**	29	30	30	29	29	30	30	28	<28
DS	6	**3**	/	6	6	5	5	7	4	5	7	<4
DIAS cons (/15)	/	/	/	**/**	/	/	**/**	**/**	15	15	**/**	<14
DIAS vow (/15)	/	/	/	/	/	/	**/**	/	15	15	**/**	<15
DIAS dia	/	/	/	/	/	/	**/**	**/**	147	80	**/**	<56
WEZT (/40)	/	/	/	**/**	/	**/**	**/**	**37**	38	39	**/**	<38
Dysarthria	−	−	−	−	−	−	−	−	−	−	−	/
FDG-PET	ATL	ATL	ATL	ATL	ATL	L ATL	ATL	ATL	ATL	/	L ATL	/
Tau CSF	/	269	/	/	**/**	/	262	**428**	/	/	267	>367
Aβ42 CSF	/	1330	/	/	/	/	733	1558	/	/	1299	<500
Neuropath	AD + TDP	/	/	/	/	/	/	/	/	/	/	/

*Note*. Norms were calculated as 2 standard deviations below the mean in the age- and education-matched group of healthy controls (*n* = 29). CPM: Raven’s progressive matrices. Dis. Dur.: disease duration (years). BNT: Boston Naming test. AAT: Akense Afasie test repetition scores part 1 to 5. DS: digit span forward. DIAS cons: Diagnostic Instrument for AOS (DIAS) repetition of consonants. DIAS vow: DIAS repetition of vowels. DIAS dia: DIAS diadochokinesis score. WEZT: grammaticality score Verbs and Sentences. FDG-PET: [18F]fluorodeoxyglucose PET, hypometabolism in the anterior temporal lobe(s)(ATL) was observed, either bilateral or on the left side (L). Cerebrospinal fluid (CSF) analysis of Aβ1-42 and total tau using ELISA. Neuropath: anatomopathological findings. SV: semantic variant of primary progressive aphasia. Values outside the normal range are in bold.

If a common temporal scaffolding mechanism structures perceptual input and speech output, perhaps speech metrics can be identified that correlate to auditory rhythmic abilities. As a candidate marker for the suprasegmental timing of speech, we calculated the pairwise variability index (PVI) of vowel nucleus duration. There is no definitive marker to diagnose AOS based on speech samples, but PVI changes have been repeatedly shown in AOS (Ballard et al., 2014; Duffy et al., 2017; Vergis et al., 2014). PVI changes presumably reflect impaired motor planning of multisyllabic strings (Ballard et al., 2014), which are more frequently impacted than single syllable words. However, the specificity of PVI to diagnose AOS remains unclear: Relevant to our study is that dysarthria is also known to impact PVI (Duffy et al., 2017).

In summary, we here examine the link between speech production and auditory rhythmic abilities interpreted in the context of a neurocomputational temporal scaffolding mechanism and aim to identify the neuroanatomical substrate of impaired rhythmic processing in NFV with AOS.

## METHODS

### Participants

Participants were recruited only if they were able to provide written informed consent. The study adhered to all requirements for the provisions of the Declaration of Helsinki (World Medical Association, Edinburgh, 2000). Approval was issued by the Ethics Committee of UZ/KU Leuven before the study commenced. PPA patients were recruited via the memory clinic of the University Hospitals Leuven. A consecutive series of 37 patients who fulfilled the international consensus criteria for PPA (Gorno-Tempini et al., 2011) enrolled for the experiment (2011–2019). The first 23 patients were described in Grube et al. (2016), and the same case numbers are used. Six patients were excluded due to hearing loss (*n* = 3); lack of ability to perform the experimental tasks due to disease severity (*n* = 2); and lack of cooperation (*n* = 1). The remaining 31 patients, who all spoke Dutch as their first language, were able to undergo the extensive testing (also performed in Dutch) and produce reliable data. Before enrollment, each patient was classified according to the 2011 recommendations (Gorno-Tempini et al., 2011). The classification relied on clinical evaluation by two neurologists with expertise in PPA. Twelve cases were classified as NFV (Table 1), 11 as the semantic variant (SV, Table 2), and 8 as the logopenic variant (LV). The LV group will not be discussed because of the smaller sample size compared to the NFV and SV groups.

AOS (defined as slow speech rate, distortions, syllable segmentation, and lengthened intersegment durations; Josephs et al., 2012) was clinically diagnosed in all NFV patients (consensus diagnosis by both neurologists). All NFV cases except case 6 clinically exhibited agrammatism at presentation, and case 6 developed agrammatism later in the disease course. Upon clinical examination, five out of the 12 NFV patients were found to have dysarthria, which is common in NFV: two cases displayed spastic dysarthria, two cases displayed hypokinetic dysarthria, and case 37 had mixed spastic-hypokinetic dysarthria. Dysarthria was moderate in cases 23, 31, and 37, as well as reduced intelligibility, in contrast to the other cases who displayed (very) mild dysarthria. Five patients (cases 20–23 and 31) also displayed clinically relevant single-word comprehension deficits early in the disease (quantified by abnormal scores on written and auditory tests of single-word comprehension; Schaeverbeke et al., 2018). Those five cases would also fit the more recently described criteria for the mixed variant (MV) subtype (Mesulam et al., 2014), which is not formally recognized in the current diagnostic classification (Gorno-Tempini et al., 2011). In cases 21 and 23, the single-word comprehension deficit was less prominent compared to the NFV characteristics. In the other cases, the comprehension deficit was deemed equally substantial. The MV subtype was described after enrollment for our study started. Hence, it is possible that some NFV cases enrolled before 2014 also meet the criteria for MV (5 NFV patients were recruited before 2014).

**Figure 1.**
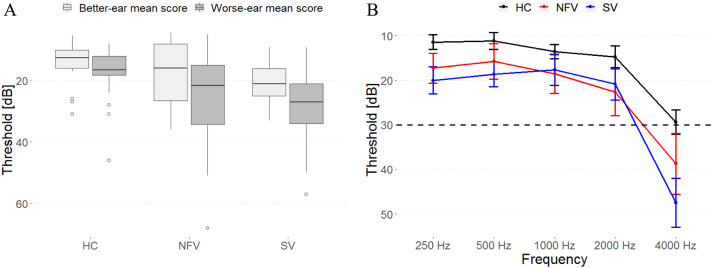
Pure-tone audiograms of all participants. (A) Mean composite ear and frequency score (250–4000 Hz) data for each participant group. (B) Mean thresholds (and standard error of the mean) for detection of tones at frequencies of 250, 500, 1000, 2000, and 4000 Hz for each participant group. HC: healthy controls. NFV: participants with non-fluent variant of primary progressive aphasia. SV: participants with semantic variant of primary progressive aphasia.

All patients received a volumetric MRI scan, and seven NFV and seven SV DTI imaging. Twenty-nine controls (15 male, age range 51–76, education 9–22 years) performed the psychoacoustic tasks, 24 received volumetric MRI, and 20 DTI imaging. Hearing sensitivity was measured in all participants using a clinical Bekesy-type audiometer for frequencies of 0.25, 0.5, 1, 2, 4, and 8 kHz on the left and right ear. Impaired pure-tone perception has been reported in NFV (Hardy et al., 2019), but here we included only participants able to detect stimuli of up to 1000 Hz below a hearing level of 30 dB on at least one side (Figure 1), because the tasks used 500 Hz pure tones. Controls, NFV, and SV were not significantly different in terms of age, sex, education, or better-ear mean score; one-way analysis of variance (ANOVA), all *p*s > 0.136.

### Behavioral Testing

Confrontation naming was tested using the Boston Naming test with Dutch norms (Mariën et al., 1998). Non-verbal executive functioning was evaluated using Raven’s Coloured Progressive Matrices. Speech repetition was assessed using the Akense Afasie test (Graets et al., 1992). Note that AOS, agrammatism, and dysarthria were diagnosed clinically prior to enrollment. To quantify the degree of AOS, the Dutch Diagnostic Instrument for AOS (Diagnostisch Instrument voor Apraxie van de Spraak; DIAS; Feiken & Jonkers, 2012) was added when it became available (for this reason it was not performed in 4 of the 12 NFV cases). The DIAS consists of vowel and consonant repetition (15 trials each) and diadochokinesis testing. During the latter task, the examiner first reads three successive alternating syllables aloud, e.g., “pa ta ka” and asks the patient to repeat these. If successful, the patient is asked to repeat it as many times as possible during a period of 8 s. The diadochokinesis severity score is the sum of correctly repeated syllables across trials. To quantify grammaticality, we used a sentence comprehension test, the Dutch verbs and sentences test (Werkwoorden en Zinnen Test; WEZT; Bastiaanse & Maas, 2000), consisting of 40 sentence-picture matching trials with active or passive sentences containing possible role reversal (e.g., “the horse was kicked by the cow”).

### Psychoacoustic Tasks

**Figure 2.**
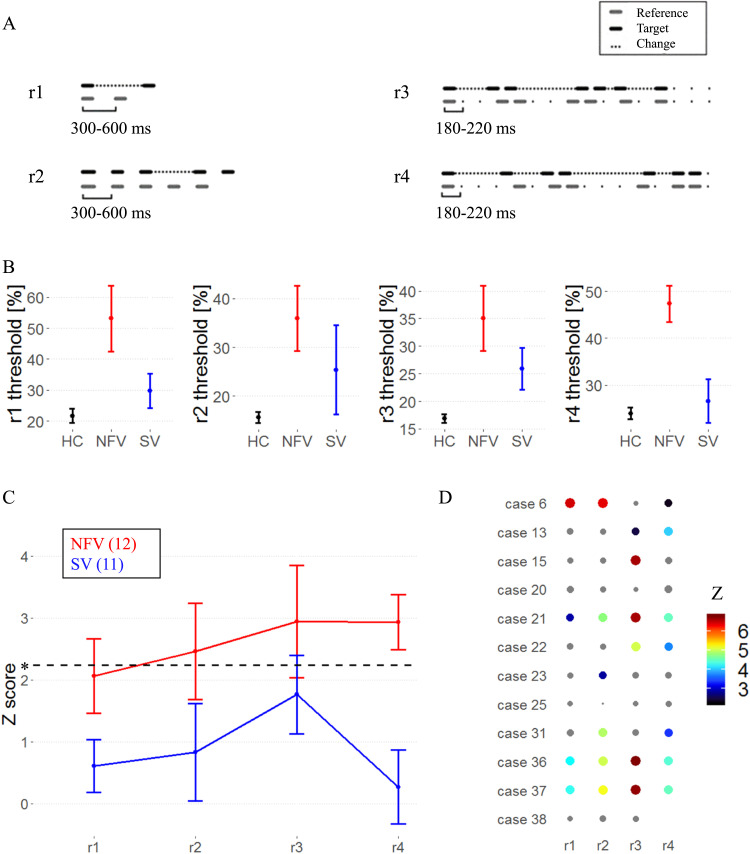
Psychoacoustic tasks. (A) Experimental design: audio samples in supplementary information. (B) Mean raw thresholds (and standard error of the mean) across controls, NFV, and SV patients. (C) Mean *z* scores (and standard error of the mean) across primary progressive aphasia (PPA) subtypes. Dotted line represents the *z* cut-off for Bonferroni-corrected *p* < 0.05. (D) *z* scores for psychoacoustic tests in NFV (case numbers refer to Table 1). The size and color of dots reflect *z* score values; nonsignificant values are gray. Missing data indicates that the patient was unable to perform the task. HC: healthy controls. NFV: participants with non-fluent variant of primary progressive aphasia. SV: participants with semantic variant of PPA.

Testing consisted of four pre-existing tasks (r1–r4; Grube et al., 2016) : the single time-interval duration discrimination task (r1), the Isochrony deviation detection task (r2), and two metrical pattern discrimination tasks (r3, r4). See Figure 2A and the additional audio files 1–4 in the supplementary information. (Supporting Information can be found at https://doi.org/10.1162/nol_a_00075). The tasks followed a two-alternative forced-choice algorithm. Participants responded verbally or by pointing to a graphical scheme. Instructions, verbal and graphic, were repeated until the participant understood the task. Five practice trials were repeated until five consecutive correct responses were recorded, and if needed, instructions were repeated and the nature of the errors was explained. If the participant indicated during the test phase that they had forgotten the instructions, then they were repeated, the practice trials run again, and the test phase then restarted.

All tasks used 500 Hz 100 ms pure tones and consisted of 50 trials. The tasks were based on a two-alternative forced-choice adaptive paradigm following a 2-down, 1-up algorithm (i.e., difficulty increasing after 2 consecutive correct responses and decreasing after every incorrect one). A larger step size was used up to the fourth reversal and after that a smaller one. The outcome measure was the threshold, calculated as the mean over the last six reversals measured with the small step size, estimating the 70.9%-correct point of the psychometric function (Levitt, 1971). The difference between the target and distractor was varied as a relative proportion of the duration or tempo of the reference. The Single time-interval duration discrimination task (r1) required participants to indicate which of the two-tone pairs contained the “longer gap.” Initially, the target was longer by 90% of the reference inter-onset-interval (depending on the trial, between 300 and 600 ms), and adaptively adjusted in steps of 12% and 6%. In the Isochrony deviation detection task (r2), participants were instructed to indicate which of two otherwise isochronous five-tone sequences contained a lengthening or “extra gap.” The reference sequence had an isochronous inter-onset-interval ranging from 300 to 600 ms. The target had one lengthened inter-onset-interval between the third and fourth tone. The initial default value of the lengthening was 60% of the inter-onset-interval, adaptively adjusted in steps of 6% and 2%. In both tasks (r1, r2), a local deviation is introduced to generate the target. As such, these tasks test the detection of lower-order differences in timing between consecutive tones.

In the metrical pattern discrimination tasks (r3, r4), participants had to decide which of three rhythmic sequences (the second or the third) of seven tones sounded “different,” based on a distortion within the rhythm. The reference sequence had a strongly (r3) or a weakly (r4) metrical beat of four evoked by the temporal spacing of the tones over 16 time units. In the strongly metrical sequence, accented tones occurred every four units, in the weakly metrical sequence, two of those were silent (Grube & Griffiths, 2009). The default initial distortion in pattern (a change in the long compared to the short intervals) was 65%, adaptively adjusted in steps of 12% and 6%. Metrical pattern discrimination (r3, r4) requires processing of the higher-order temporal structure of the stimuli, since global deviations distributed across the sequence need to be detected. Typical syllable rates in Dutch (the native language of the participants) are 4–5 syllables per second (period 200–250 ms), which is close to the tempi used in our tasks.

### Statistical Analysis of Psychoacoustic Tasks

Depending on the distribution, outcome measures were log-transformed to allow for parametric analysis at the group level. At the individual level, each patient’s performance on the psychoacoustic tasks was analyzed in comparison to the group by using a modified Crawford *t* test (Crawford & Garthwaite, 2007). For the comparison between each patient and the controls, to facilitate comparison between tasks and to enable Bonferroni correction, the exact *p* values (estimated percentiles) calculated according to Crawford and Garthwaite were transformed into normalized *z* scores using the standard normal cumulative distribution function. The significance threshold was set to a one-tailed significance level of *p* < 0.05, Bonferroni-corrected for the number of tests (*n* = 4 for the psychoacoustic tasks, one-tailed since the a priori hypothesis is that NFV would perform worse). We compared the psychoacoustic thresholds between NFV and SV using a Student’s *t* test (one-tailed significance level of *p* < 0.05, effect size: Cohen’s *d* with Hedges correction for small samples).

### Connected Speech Analysis

To obtain a tentative measure of the suprasegmental timing of speech, we determined the normalized PVI for vowel nucleus duration in connected speech samples using Praat 6.1.02 (https://www.fon.hum.uva.nl/praat/). The samples consisted of a 2-minute “Cookie Theft Scene” description (20 controls, 11 NFV, 9 SV, samples in Dutch). For every participant, the median PVI (Ballard et al., 2014) was determined for polysyllabic words with a strong-weak stress pattern (e.g., COO-kie) and for words with a weak-strong stress pattern (e.g., out-DA-ted). PVI was calculated following the procedure outlined in Ballard et al. (2014) and Duffy et al. (2017), equaling 100 × (d1 − d2) / [(d1 + d2) / 2], where d1 and d2 are the durations of the first and second vowel. False starts, which may occur in the speech of NFV, were ignored for the purpose of the calculations. Normalization corrects for a difference in speech rates. PVI values closer to zero are consistent with relatively equal stress between the first two vowels of a word (Ballard et al., 2014). We also report absolute vowel durations for each subgroup to provide more insight into the PVI values, as well as the results per patient group, since PVI changes may be language-specific (Ballard et al., 2014). For NFV, SV, and controls, we correlated PVI to the psychoacoustic tasks to test the link between auditory rhythmic abilities and speech production (significance level of *p* < 0.05). In an exploratory analysis, we also correlated the PVI metrics demonstrating a correlation to the psychoacoustics tasks to the white matter metrics.

### Acquisition of MRI Data

Twenty-three patients (12 NFV, 11 SV) and 24 controls received a high resolution T1-weighted structural MRI. All controls and 13 patients were scanned on a 3T Philips Intera system equipped with an 8-channel receive-only head coil (SENSitivity Encoding head coil). Ten patients were scanned on a 3T Philips Achieva dstream scanner equipped with a 32-channel head volume coil. An identical 3D turbo field echo sequence was used on both systems (coronal inversion recovery prepared 3D gradient-echo images, inversion time (TI) 900 ms, shot interval = 3,000 ms, echo time (TE) = 4.6 ms, flip angle 8°, 182 slices, voxel size 0.98 × 0.98 × 1.2 mm^3^). The diffusion weighted images consisted of 45 directions of diffusion weighting with b = 800 as well as 1 non-diffusion weighted image (B0), acquired in the axial plane, with isotropic voxel size of 2.2 mm, TR 9,900 ms, TE 90 ms, flip angle 90°, fold over direction AP, fat shift direction A (anterior), in-plane parallel image acceleration (SENSE) factor 2.5.

### Deformation-Based Morphometry

DBM was performed using the CAT12 toolbox (https://www.neuro.uni-jena.de/cat), an extension of SPM12 (https://www.fil.ion.ucl.ac.uk/spm). Segmentation was performed in CAT12 using a default tissue probability map. Local adaptive segmentation was used at default strength (medium) and diffeomorphic anatomical registration through exponentiated lie algebra (DARTEL) was used for registration to the default template (IXI555_MNI152). Voxel size for normalized images was set at 1.5 mm (isotropic) after internal resampling at 1 mm. Local deformations were estimated using the Jacobian determinant, while ignoring the affine part of the deformation field. Thus, additional correction for total intracranial volume is not required (Gaser & Kurth, 2019). Images were smoothed using a 8 × 8 × 8 mm^3^ Gaussian kernel. Jacobian maps of controls and both PPA groups were compared using a one-way between-subject ANOVA. Multiple linear regression was used to correlate the psychoacoustic tasks (r1–r4) at the individual level to the Jacobian maps within each PPA subtype. Scanner type and age were introduced as nuisance variables in all analyses. Threshold of significance was set at voxel-level uncorrected *p* < 0.001 and cluster-level family-wise error (FWE) corrected *p* < 0.05 (Grube et al., 2016; Poline et al., 1997). The resulting volume of interest was mirrored to explore contralateral effects. To determine whether the observed DBM result was related to grey or white matter involvement, a supplementary whole-brain VBM analysis using respectively segmented grey and white matter maps was performed in which total intracranial volume (TIV) was also added as a nuisance variable.

### Diffusion Tensor Imaging

Diffusion images were preprocessed and analyzed using the KU Leuven neuroimaging suite (KUL_NIS; Radwan & Sunaert, 2022), which relies on MRTrix3 (Tournier et al., 2012, 2019); FSL (Jenkinson et al., 2012; Smith et al., 2004); and ANTs (Tustison et al., 2021). The preprocessing pipeline included the following steps: First, the data were converted to the brain imaging data structure convention (BIDS), then to MIF using mrconvert. Diffusion preprocessing included denoising using dwidenoise (Veraart et al., 2016); echo planar imaging (EPI) distortion correction was done using Synb0-DisCo (Schilling et al., 2019) and FSL topup (Andersson et al., 2003). Correction for subject motion, and eddy current artefacts was performed using MRTrix3 dwifslpreproc, which relies on FSL eddy (Andersson & Sotiropoulos, 2016).

This step was followed by imaging bias correction with dwibiascorrect. DTI (Basser et al., 1994a, b) and CSD (Tournier et al., 2007) estimation was done using MRTrix3 dwi2tensor and dwi2response. White matter response functions were averaged over the whole group and the average response functions were used to calculate fiber orientation distribution (FOD) maps for every subject using dwi2fod. FreeSurfer’s recon-all (Fischl, 2012), and the multi-scale brain parcellator (Tourbier et al., 2020) were used for parcellating the 3D T1-weighted images, based on the Desikan-Killiany atlas (Desikan et al., 2006) and the Lausanne parcellation (Cammoun et al., 2012).

Next, we used the KU Leuven Fun-With-Tracts package (KUL_FWT; Radwan et al., 2022) for calculating DTI scalar maps, and parcellation-based probabilistic CSD tractography using second-order integration over orientation distributions (iFOD2; Tournier et al., 2010). The bundle-specific approach of KUL_FWT was used for frontal Aslant tract tractography: Tractograms were initially generated with 8,000 streamlines. We used the IFG pars triangularis and pars opercularis labels from aparc+aseg maps generated by FreeSurfer, and the superior frontal gyrus 5 & 6 labels from the scale-3 maps generated by the multi-scale brain parcellator as the first and second inclusion regions, respectively. The whole corpus callosum, brainstem, and orbitofrontal cortex labels from the aparc+aseg were used as exclusion regions.

Smoothed fractional anisotropy (FA) and mean diffusivity (MD) maps were compared between controls, NFV, and SV using a between-subject ANOVA (threshold same as DBM/VBM). Scanner type, TIV, and age were introduced as nuisance variables. A template for the left Aslant tract was generated for healthy controls using the 75% overlap threshold (Catani et al., 2013). FA and MD of the left Aslant tract were extracted for each patient by averaging values from all voxels included in this template (Catani et al., 2013). We compared the FA and MD between NFV and SV by means of a Student’s *t* test (one-tailed *p* < 0.05). FA and MD were correlated to rhythmic abilities to confirm the DBM findings (one-tailed *p* < 0.05).

## RESULTS

### Psychoacoustic Tasks

Two tasks (single time-interval duration discrimination (r1) and isochrony deviation detection (r2); see Figure 2A and additional audio files 1–2) probed the detection of local deviations of temporal structure. Two tasks (metrical pattern discrimination tasks using a strongly (r3) or weakly (r4) metrical reference; see Figure 2A and additional audio files 3–4) probed the detection of higher-order deviations of temporal structure. The latter tasks (r3, r4) measure the “rhythmic abilities” of each participant. Based on the suprasegmental abnormalities found in the patients’ speech, we postulate that the tasks indexing suprasegmental timing (r3, r4) will be most impaired in NFV with AOS.

In a group-level analysis, performance on the psychoacoustic tasks was poorer in NFV compared to controls: Mean *z* scores were significantly increased (higher individual psychoacoustic thresholds equaling poorer abilities to detect deviations) in NFV for discrimination of strongly metrical sequences (r3, mean *z*: 2.94), discrimination of weakly metrical sequences (r4, mean: 2.93), and isochrony deviation detection (r2, mean: 2.46) (Figure 2B–C). We compared the psychoacoustic test scores between the NFV and SV. This resulted in significantly poorer scores in NFV for the discrimination of weakly metrical sequences (r4, *p* = 0.001, Hedges’ *g*: 1.48) (Figure 2B–C). At the individual level, deficits were observed mainly in NFV patients (*z* > 2.24) (Figure 2D). The weakly metrical pattern discrimination task (r4) demonstrated a significant impairment in 7 NFV (Figure 2D) and 2 SV patients. Similarly, strongly metrical pattern discrimination (r3) was impaired in 6 NFV (Figure 2D) and 4 SV, as well as isochrony deviation detection (r2) in 6 NFV and 2 SV patients. Single time-interval discrimination (r1) was impaired in just 4 NFV and 1 SV patients. In summary, 9/12 NFV cases and 4/11 SV cases were impaired in one or more of the tasks. No correlation was found between better-ear hearing levels and psychoacoustic scores in patients (all *p*s > 0.220).

### Correlation of Psychoacoustic Tasks With Speech Timing

**Figure 3.**
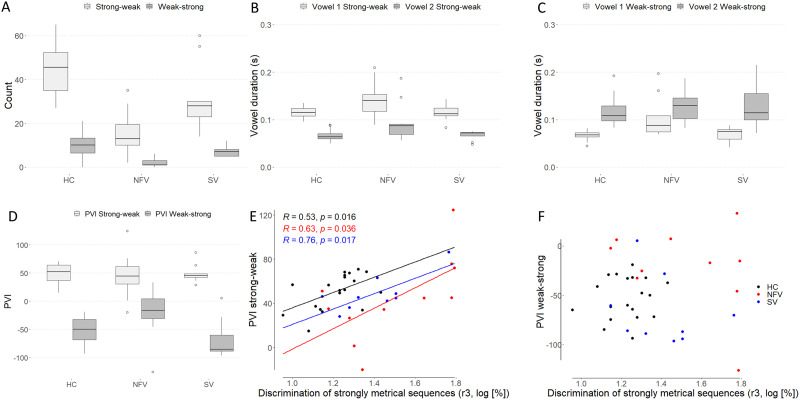
Suprasegmental timing of speech. (A) Frequency of words with a strong-weak pattern and a weak-strong pattern in the three participant groups. (B–C) Vowel durations for words with (B) a strong-weak pattern and (C) a weak-strong pattern. (D) PVI of words with a strong-weak and a weak-strong pattern. (E–F) Correlation of PVI for (E) strong-weak and (F) weak-strong words and strongly metrical sequence thresholds (r3, log-transformed, %); regression line indicates a significant correlation at the subgroup level. PVI: pairwise variability index. HC = healthy controls. NFV: participants with non-fluent variant of primary progressive aphasia. SV: participants with semantic variant of primary progressive aphasia.

To determine PVI as a proxy for suprasegmental timing of speech, vowel duration was first calculated for all polysyllabic words with a strong-weak pattern (stress on the first syllable) and a weak-strong pattern (stress on the second syllable). As expected in Dutch, participants generated more words with a strong-weak pattern compared to a weak-strong pattern (Figure 3A). Lengthening of the first and second vowel (absolute duration) was observed for words with a strong-weak pattern in NFV compared to controls (1st vowel: one-way ANOVA *F*(2, 37) = 4.17, *p* = 0.015; 2nd vowel: ANOVA *F*(2, 37) = 5.45, *p* = 0.008; in both cases post hoc testing demonstrated differences between the NFV with AOS and the control group; Figure 3B). Lengthening of the first vowel was observed for words with a weak-strong pattern in NFV compared to controls (one-way ANOVA *F*(2, 35) = 5.11, *p* = 0.011; Figure 3C). A trend was observed for between-group differences of the duration of the second vowel in these words (one-way ANOVA *F*(2, 35) = 2.9, *p* = 0.068; Figure 3C).

PVI reflects the relative duration of the stressed versus unstressed syllable. PVI values were closer to zero (relatively equal stress) for words with a weak-strong stress pattern in NFV compared to controls and SV (one-way ANOVA *F*(2, 35) = 5.37, *p* = 0.009; Figure 3D). No significant between-group differences were found for strong-weak words. Across all participants, PVI for strong-weak words correlated with the psychoacoustic threshold for strongly metrical pattern discrimination (r3) (*r* = 0.444, *p* = 0.004; Figure 6E). In an analysis per subgroup, this correlation was found for NFV (*r* = 0.634, *p* = 0.036), SV (*r* = 0.761, *p* = 0.017), and controls (*r* = 0.531, *p* = 0.016). This means that participants with poorer perceptual rhythmic abilities displayed greater duration differences between the first and second vowels of words with a strong-weak stress pattern. No correlation was found with any of the other psychoacoustic tasks (r1, r2, r4), and no correlation was found with the PVI for weak-strong words (all *p* > 0.1, Figure 3F).

### White Matter Changes: Deformation-Based Morphometry

**Figure 4.**
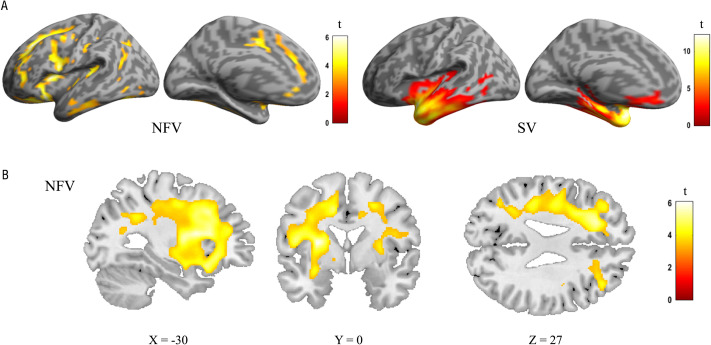
Deformation-based morphometry (DBM) analysis: comparison of controls, the NFV, and SV patients. (A) Renderings shows atrophy of 12 NFV and 11 SV compared to 24 controls (cluster-level FWE-corrected *p* < 0.05). (B) Slices in NFV. NFV: participants with non-fluent variant of primary progressive aphasia. SV: participants with semantic variant of primary progressive aphasia.

**Figure 5.**
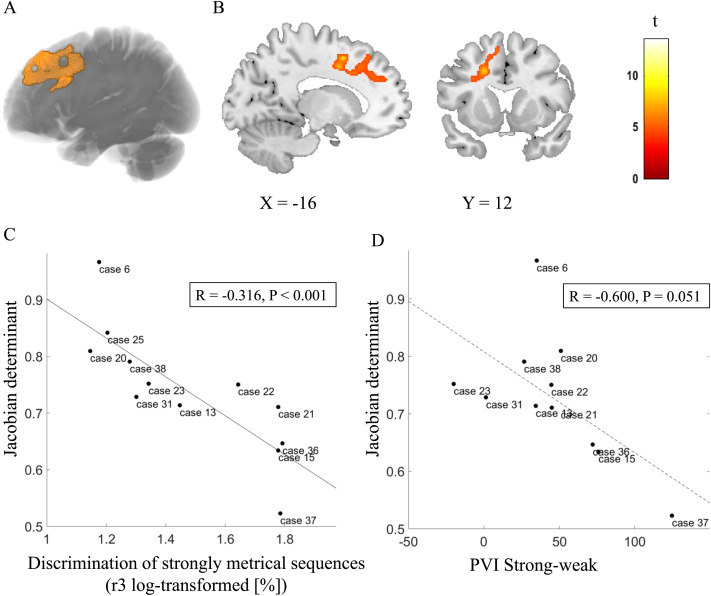
Deformation-based morphometry (DBM) of rhythmic abilities in NFV cases. (A) Rendering of the DBM result (orange) projected onto structural template. (B) Slices. (C) Correlation between volume loss and strongly metrical sequence thresholds (r3, log-transformed, %) in NFV patients in the region of interest (A–B), exploratory plot for illustrative purposes. (D) Correlation between volume loss and PVI for words with a strong-weak pattern in NFV patients in the region of interest (A–B) (dotted regression line since there was a trend for significance). Case numbers refer to Table 1. PVI: pairwise variability index.

A whole-brain DBM analysis was conducted to characterize the atrophy patterns for each PPA group and to test whether rhythmic processing correlated with atrophy. The expected atrophy patterns per PPA subgroup were observed using DBM (Figure 4). In the NFV group, atrophy was observed mainly in the frontal lobes compared to controls, with a left-sided predominance (Figure 4A–B). In the SV group, atrophy was localized to the anterior temporal lobes (Figure 4A). In the NFV group, voxel-wise multiple linear regression showed that the strongly metrical rhythm discrimination task (r3) negatively correlated with volume changes in the left frontal white matter (Montreal Neurological Institute (MNI) = −20, 20, −36; −17, 8, 48; −9, 39, 50; k_E_ 2,426 voxels, *z* score: 4.92) (Figure 5A–B). This negative correlation indicates that poorer rhythmic abilities (i.e., larger psychoacoustic thresholds) were linked to more atrophy. For illustrative purposes, we plotted the individual NFV thresholds for the strongly metrical discrimination task (r3) versus volume loss in this region (*r* = −0.316, *p* < 0.001) (Figure 5C). The correlation between the PVI for words with a strong-weak pattern and volume loss in this region showed a trend for significance (*r* = −0.600, *p* = 0.051) (Figure 5D). No correlation was observed with the strongly metrical discrimination task (r3) in the right-sided homologue volume of interest (VOI) (*r* = −0.347, *p* = 0.267). Finally, DBM analysis yielded no other significant correlations with the psychoacoustic tasks in the NFV or SV subgroups. The additional VBM analysis using segmented white matter maps of the NFV patients confirmed that the loss of volume was related to white matter changes: When correlating the strongly metrical discrimination task (r3) with white matter intensity, 2 clusters survived the preset significance threshold and the largest cluster (peak coordinates MNI = −26, 18, 30; k_E_ 232 voxels, *z* = 3.74) overlapped with the result of the DBM analysis (Figure 5A–B). In the VBM analysis using grey matter maps, no clusters survived the preset threshold.

### White Matter Changes: Diffusion Tensor Imaging

**Figure 6.**
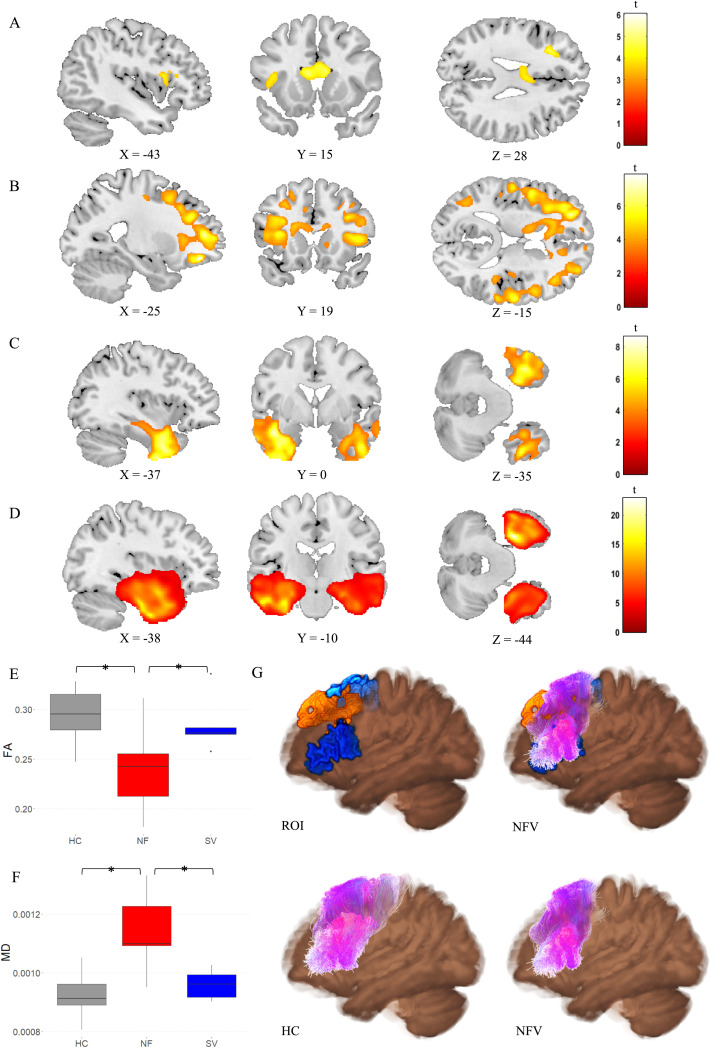
Diffusion tensor imaging (DTI) metrics. (A) Fractional anisotropy (FA) and (B) Mean diffusivity (MD) in NFV patients versus controls (cluster-level FWE-corrected *p* < 0.05). (C) FA and (D) MD in SV patients versus controls (cluster-level FWE-corrected *p* < 0.05). Comparison of (E) FA and (F) MD in the left frontal Aslant tract between the NFV and SV subtypes and healthy controls (HC). (G) 3D visualization of the left Aslant tract projected on the average FA map—Top left: seed regions (blue) and result of deformation-based morphometry (DBM) (orange); Top right: seed regions (blue) and result of DBM (orange) combined with Aslant tract streamlines of NFV patients (purple); Bottom left: streamlines of controls (purple); Bottom right: streamlines of NFV patients (purple). ROI: region of interest. HC: healthy controls. NFV: participants with non-fluent variant of primary progressive aphasia. SV: participants with semantic variant of primary progressive aphasia.

A whole-brain comparison between NFV, SV, and controls showed reduced FA in NFV in the left inferior frontal region, the corpus callosum, and the anterior cingulate (Figure 6A). MD was widely increased in NFV, with a predominance in both frontal lobes (Figure 6B). In SV, FA was reduced and MD was increased in both anterior temporal lobes (Figure 6C–D). We then compared FA and MD between NFV and SV specifically within the template of the left Aslant tract derived from the controls. FA was lower in NFV compared to SV (*p* = 0.038, Hedges’ *g*: −1.17; Figure 6E), and MD was higher in NFV compared to SV (*p* = 0.003, Hedges’ *g*: 1.85; Figure 6F). Across all participants, FA and MD in the left Aslant tract correlated to the performance on the strongly metrical rhythm discrimination task (r3) (FA: *r* = −0.535, *p* = 0.001; MD: *r* = 0.652, *p* < 0.001). This means that FA was lower and MD was higher when performance on the metrical rhythm discrimination task (r3) was weaker. Within the relatively small NFV group, a trend in the expected direction was observed when correlating FA and MD in the left Aslant tract with the performance on the strongly metrical rhythm discrimination task (r3) (FA: *r* = −0.670, *p* = 0.099; MD: *r* = 0.745, *p* = 0.053). In the contralateral homologue VOI, no correlation was observed (FA: *r* = −0.517, *p* = 0.235; MD: *r* = 0.245, *p* = 0.597). Neither FA nor MD in the left Aslant tract correlated with performance on any other psychoacoustic task in NFV (r1, r2, r4, all *p*s > 0.271), but there was a correlation between the DTI metrics and PVI for words with a strong-weak pattern (FA: *r* = −0.791, *p* = 0.034; MD: *r* = 0.905, *p* = 0.005). Visual inspection of the left Aslant tract in NFV showed that this tract overlapped with the region where there were white matter volume changes identified by DBM (Figure 6G).

## DISCUSSION

In patients with NFV and AOS, we investigated the neural correlate of impaired auditory rhythmic processing. In a group of 12 consecutive cases with NFV, we confirmed that rhythmic abilities are overall poorer compared to controls and patients with SV (Grube et al., 2016). Behaviorally, we observed a correlation between rhythmic abilities and a marker for the suprasegmental timing of speech for NFV as well as for SV and healthy controls, in agreement with a coupling between auditory perception and speech production. DBM demonstrated that atrophy in the left frontal lobe correlated with the individual patients’ rhythmic abilities. We complemented DBM with DTI to provide an independent measure of white matter changes. DTI confirmed a correlation between the left Aslant tract MD and FA and rhythmic abilities. Given the prior work implicating the left Aslant tract in non-fluent speech and AOS (Canu et al., 2019; Catani et al., 2013; Mandelli et al., 2014), our results indicate it may be a part of common anatomical substrate for rhythmic abilities and speech production. Whilst our findings are correlational, the results for speech timing complement the two independent white matter metrics. Our findings strengthen the evidence base for a well-defined neurocomputational mechanism of temporal scaffolding linking perception and speech production. In NFV, the concept of impaired temporal scaffolding ties both core auditory deficits and AOS to left frontal atrophy.

Although four psychoacoustic tasks were performed, both the marker for the suprasegmental timing of speech and left frontal lobe atrophy in NFV were linked to impaired performance on the strongly metrical rhythm discrimination task (r3). This task is conceptually different from the single time-interval duration discrimination task (r1) and the isochrony deviation detection task (r2): Determining the metricality of a tone sequence (r3) requires processing of the higher-order temporal structure determined by the grouping of salvos of notes that induce the sense of a regularly occurring metrical “beat” (Grube & Griffiths, 2009). Metricality-based rhythm discrimination (r3) necessitates detecting global deviations distributed across the entire sequence. Our results are in agreement with prior work: The detection of temporal changes between syllables was more impaired in PPA compared to controls when stimuli contained a higher number of syllables (Rohrer et al., 2012). In contrast, the single time-interval duration discrimination task (r1) and the isochrony deviation detection task (r2) test lower-order differences in timing in a simple isochronous sequence based on a local deviation. The weakly metrical rhythm discrimination task (r4) is more challenging as it does not rely on a clear metrical beat (Grube & Griffiths, 2009) (higher thresholds for r4 versus r3 in controls, *p* < 0.001). Perhaps domain-general processes play a larger role in this task compared to the strongly metrical rhythm discrimination task (r3), but additional manipulations are required to support this hypothesis. Specifically, the relation between rhythm processing and working memory deficits in NFV should be investigated more thoroughly using tasks indexing auditory working memory, phonological working memory, and working memory tasks not grounded in the auditory modality. Decreased auditory working memory (measured by digit span) is usually observed in NFV and has been linked to agrammatism (Rohrer et al., 2010). In our NFV cases, we did not find a strong correlation between metrical pattern discrimination (r3, r4) and digit span (both *p* > 0.093). The prior musical experience of the individual participants may also have influenced their performance on the psychoacoustic tasks. Keeping in mind the labor-intensive administration of our tasks, we reached a considerable albeit modest sample size. We acknowledge that further validation of our findings requires a larger multicenter sample given the relative rarity of PPA.

Using two independent white matter metrics, we identified an overlapping white matter substrate in the left frontal lobe that may play a role in both rhythmic abilities and speech production and is thus a candidate component of the temporal scaffolding mechanism. We observed white matter degeneration close to the SMA. The Aslant tract connects the superior frontal gyrus/SMA to the IFG, cortical regions that have previously been implicated in rhythm processing (Poeppel & Assaneo, 2020). SMA was linked to auditory rhythmic processing (Hardy, Agustus, Marshall, Clark, Russell, Bond, et al., 2017) and AOS (Tetzloff et al., 2018; Whitwell et al., 2013) to NFV. IFG plays a role in speech rhythm (Long et al., 2016) and synchronizing speech to external auditory stimuli (Assaneo et al., 2019). The correlation between rhythmic abilities and white matter integrity in the left frontal lobe aligns with the contemporary view that speech rhythm production and perception are sustained by a left hemispheric network rather than a single cortical region (Mandelli et al., 2016; Poeppel & Assaneo, 2020). Besides the Aslant tract, other white matter tracts, e.g., tracts connecting to the auditory cortices (Assaneo et al., 2019), may also play an important role in this network. Accordingly, the DBM analysis also demonstrated that the region in which atrophy correlated to rhythmic abilities extends beyond the boundaries of the left Aslant tract. While we focus here on white matter damage, the existing literature suggests that cortical damage also contributes to abnormal speech rhythm production and perception in NFV (Hardy, Agustus, Marshall, Clark, Russell, Bond, et al., 2017). While VBM did not detect a correlation between cortical atrophy and rhythmic abilities in our NFV group, this does not preclude functional abnormalities at the cortical level. The unique contributions of grey and white matter to rhythmic processing require further study in a larger cohort.

An important consideration is whether the white matter changes reflect tau pathology, which is found in up to 88% of NFV patients (Spinelli et al., 2017). In particular, DTI metrics have been put forward as a marker of tauopathy and other proteinopathies (Downey et al., 2015; Mahoney et al., 2013). DTI imaging is sensitive to changes caused by tau pathology at the single-subject level (Sajjadi et al., 2013), presumably because of underlying glial pathology (Forman et al., 2002), e.g., by myelin injury or changes in other structures that affect water diffusion (Galantucci et al., 2011). Here, we performed DTI imaging in a small subset of the study population (7 NFV, 7 SV, and 20 controls) and because of the small sample size, we interpret these preliminary findings with appropriate caution. Diffusion MRI and fiber tractography are known to have a number of methodological drawbacks: The ones most relevant to our work are volume averaging and lack of accuracy in voxels with complex fiber architecture such as the capsules (internal, external. and extreme), as well as the centrum semiovale. We attempted to maximize the accuracy of voxel-wise comparisons by relying on CSD iFOD2 tractography, and by using streamline filtering to minimize false positive streamlines. Our preliminary DTI results are in alignment with prior work in PPA (Powers et al., 2013): MD changes were more pronounced than FA changes in NFV patients (Whitwell et al., 2010). In our study, neuropathological data is lacking for most patients, thus prohibiting us from making strong claims in relation to pathology. We would not advocate linking tauopathy to a simple DTI parameter. Rather, our findings advance the broader characterization of the possible disease-specific involvement of white matter tracts. Our results align with the “molecular nexopathy” paradigm (Warren et al., 2013): The left frontal network containing IFG and SMA as nodes demonstrates a selective vulnerability to tau protein, which could spread locally through the left Aslant tract in a prion-like fashion. Even if certain proteinopathies are strongly linked to predictable phenotypes of network disruption, the molecular nexopathy paradigm does not propose complete specificity. Accordingly, we also observed impaired perceptual rhythmic abilities in some SV patients. As the disease progresses, PPA subtypes exhibit convergence of their atrophy patterns (Bruffaerts et al., 2020; Leyton et al., 2019), even though the underlying neuropathology is different. This convergence of atrophy patterns may explain that some SV demonstrated impaired rhythmic abilities.

In an attempt to understand how impaired auditory rhythmic processing relates to AOS, we correlated each participant’s rhythmic abilities to a suprasegmental marker of speech timing, the PVI. Interestingly, we observed a correlation between speech timing for strong-weak words and metrical pattern discrimination ability in all three participant groups. Such a coupling aligns with the temporal scaffolding hypothesis because it supports the idea that both speech production and perception tap into a common neural mechanism sustaining rhythm processing at the input and output level. The fact that the coupling even holds in NFV patients further supports this notion. At the segmental level, we observed that the first and second vowel duration are both longer in words with a strong-weak pattern in NFV compared to controls. However, this did not lead to significant changes to the speech rhythm properties in the relative sense at the suprasegmental level, which replicates the findings of Vergis et al. (2014). As the dominant rhythmic structure, the production of words with a strong-weak pattern appears to be relatively spared in AOS in German (Aichert et al., 2016) and English (Bailey et al., 2019). We showed here that this is also the case in Dutch. We moreover observed a positive correlation between the suprasegmental timing of speech (reflecting the contrast between the length of the first stressed versus second unstressed vowel) and rhythmic abilities in all groups for strong-weak words. Hypothetically, increased contrast in these spoken words co-occurring with poorer rhythmic abilities may be interpreted as a “coarser” or more un-even resolution of the temporal scaffolding system and may give rise to an overall slower, and less fluent production of speech. In other words, the more accurate the system, the more even the syllables are being produced in words with strong-weak pattern, while pathology results in abnormal lengthening of the first, strong syllable. For words with a weak-strong pattern, we replicated the lower PVI values typically observed in AOS (Ballard et al., 2014; Duffy et al., 2017) and also observed abnormal lengthening of the first vowel in these words. Our behavioral findings cannot explain the full extent of speech abnormalities in AOS and evidence the need for automated, broadly validated speech-based markers in PPA.

We opted here to study the PVI for duration because of its intuitive link to suprasegmental timing, but it is clearly an imperfect marker of AOS and speech rhythm. Alternative markers (e.g., PVI for intensity/fundamental frequency, measures of silence duration or variability) may provide additional information. We acknowledge that we could not in our analysis take into account the position of the word in the sentence or other word characteristics such as word length, which may also influence the timing of speech (Aichert et al., 2016; Bailey et al., 2019; Vergis et al., 2014). Additional variability was possibly introduced by using connected speech samples resulting in different outputs across participants, while other studies used tasks that elicited an identical response from all participants. The presence of moderate dysarthria (with impact on intelligibility) in 3 out of 12 NFV cases may have also affected our marker of speech timing (Duffy et al., 2017). While PVI is a sensitive measure to identify AOS in NFV (Ballard et al., 2016), PVI should be considered an aspecific marker of motor speech impairment. Hence, when the PVI is abnormal in a patient with both dysarthria and AOS, it is not straightforward to pinpoint which motor speech abnormality drives the changes in the PVI (Duffy et al., 2017). Ideally, AOS should be studied using the PVI in patients without co-occurring dysarthria. Whilst our results can thus not be conclusive, they build on and expand findings from the existing literature on AOS.

Finally, we acknowledge that even though all our NFV cases exhibited clear AOS, the clinical picture included other symptoms often observed in NFV such as agrammatism, dysarthria, or extrapyramidal signs. We cannot quantify how these findings relate to the observed white matter changes. Ideally, our results should be replicated in patients with isolated AOS, for instance, in patients with primary progressive AOS (ppAOS) (Josephs et al., 2012), but this phenotype is even more rare than NFV (Duffy et al., 2021). Neuroimaging analyses of patients with ppAOS are in agreement with our results: Reduced FA was observed in the left SMA in ppAOS (Utianski et al., 2018), and functional connectivity analysis demonstrated that SMA is disconnected from the speech and language network in ppAOS (Botha et al., 2018). The atrophy pattern germane to ppAOS differs from the atrophy pattern reported in NFV without AOS, with bilateral volume loss more anteriorly in the frontal lobes compared to ppAOS and also in the left temporal lobe (Tetzloff et al., 2019). Lastly, an important non-neurodegenerative cause of AOS is stroke. Lesion mapping in stroke patients with AOS demonstrated maximal overlap in the left (pre)motor cortices and adjacent white matter (Graff-Radford et al., 2014), which coincides with our neuroimaging findings in white matter. Furthermore, using resting-state fMRI abnormal functional connectivity of the left premotor cortex was observed in stroke patients with AOS compared to those without AOS (New et al., 2015), indicative of white matter damage in the left frontal lobe in the patients with AOS. In summary, the regions identified as neural correlates of rhythmic processing in our study are consistent with prior neuroimaging studies on the neural correlates of AOS.

### Conclusions

Co-occurring impaired rhythmic abilities and AOS in NFV prompted our search for the neuroanatomical correlate of rhythmic processing in NFV. Our DBM and DTI findings in NFV showed concordant evidence that rhythmic abilities correlate with left frontal white matter atrophy, overlapping with the substrate for AOS. Overall, we provided initial evidence for a common neurocomputational mechanism for speech production and perception with its neural basis located in the left frontal lobe. Future studies should target larger multicentric patient cohorts with pure AOS to characterize this mechanism in greater detail. A better understanding of the neurobiological link between speech production and perception may contribute to the development of tailored rehabilitation strategies (Ballard et al., 2015).

## ACKNOWLEDGMENTS

The authors thank Bruno Bergmans, MD, PhD, Charlotte Swinnen, MD, Anne Sieben, MD, PhD, and Yolande A. Pijnenburg, MD, PhD, for the referral of patients. We thank Emma Luckett, MSc, for copyediting. RB is a senior and JS is a junior postdoctoral fellow of the Research Foundation Flanders (FWO).

## FUNDING INFORMATION

Rose Bruffaerts, Fonds Wetenschappelijk Onderzoek (https://dx.doi.org/10.13039/501100003130), Award ID: 12I2121N. Rik Vandenberghe, Fonds Wetenschappelijk Onderzoek (https://dx.doi.org/10.13039/501100003130), Award ID: G0925.15. Rik Vandenberghe, Belgian Federal Science Policy Office (https://dx.doi.org/10.13039/501100002749), Award ID: 7/11. Rik Vandenberghe, Onderzoeksraad, KU Leuven (https://dx.doi.org/10.13039/501100004497), Award ID: OT/12/097, C14/17/108.

## AUTHOR CONTRIBUTIONS

**Rose Bruffaerts**: Conceptualization: Equal; Data curation: Equal; Formal analysis: Lead; Funding acquisition: Equal; Investigation: Equal; Methodology: Equal; Project administration: Equal; Supervision: Equal; Validation: Equal; Visualization: Lead; Writing – original draft: Lead. **Jolien Schaeverbeke**: Data curation: Equal; Formal analysis: Supporting; Investigation: Equal; Project administration: Equal; Validation: Equal; Writing – review & editing: Equal. **Ahmed Radwan**: Formal analysis: Equal; Methodology: Equal; Software: Equal; Visualization: Supporting; Writing – review & editing: Equal. **Manon Grube**: Conceptualization: Equal; Formal analysis: Supporting; Methodology: Equal; Writing – review & editing: Equal. **Silvy Gabel**: Data curation: Supporting; Formal analysis: Supporting; Investigation: Equal; Project administration: Supporting; Writing – review & editing: Supporting. **An-Sofie De Weer**: Data curation: Supporting; Formal analysis: Supporting; Investigation: Equal; Project administration: Supporting; Writing – review & editing: Supporting. **Eva Dries**: Data curation: Supporting; Formal analysis: Supporting; Investigation: Equal; Writing – review & editing: Supporting. **Karen Van Bouwel**: Data curation: Supporting; Formal analysis: Supporting; Investigation: Equal; Writing – review & editing: Supporting. **Timothy D. Griffiths**: Conceptualization: Equal; Methodology: Equal; Writing – review & editing: Equal. **Stefan Sunaert**: Formal analysis: Equal; Methodology: Equal; Resources: Equal; Software: Equal; Writing – review & editing: Equal. **Rik Vandenberghe**: Conceptualization: Equal; Data curation: Supporting; Funding acquisition: Equal; Project administration: Equal; Resources: Equal; Supervision: Equal; Validation: Equal; Writing – review & editing: Equal.

## TECHNICAL TERMS

Primary progressive aphasia (PPA): A heterogeneous group of neurodegenerative diseases presenting with prominent language deficits.
Deformation-based morphometry (DBM): A neuroimaging method used to detect volume loss across the whole brain.
Diffusion Tensor Imaging (DTI): A neuroimaging technique to evaluate white matter and more specifically to examine white matter fiber tracts.
