# Supplementary material for: Left Frontal White Matter Links to Rhythm Processing Relevant to Speech Production in Apraxia of Speech
Source: Neurobiol Lang (Camb). 2022 Sep 22;3(4):515–37. doi: 10.1162/nol_a_00075 (PMC10158569; doi:10.1162/nol_a_00075)
Supplement: Supplementary file 1 [file nol-3-4-515-s001.zip › nol_a_00075-Bruffaerts-supps/supplementary.docx]

**Supplementary of the manuscript “Left frontal white matter links to rhythm processing relevant to speech production in apraxia of speech” by Bruffaerts et al.**

Each psychoacoustic task is exemplified by an audio file containing three trials. A description of the tasks can be found in the method section and a schematic overview is shown in Fig 2A.

Task_r1.wav: ‘Single time-interval duration discrimination’ task (r1)

Task_r2.wav: ‘Isochrony deviation detection’ task (r2)

Task_r3.wav: ‘Metrical pattern discrimination’, strongly metrical beat (r3)

Task_r4.wav: ‘Metrical pattern discrimination’, weakly metrical beat (r4)
